# Pulsed Optically Pumped Magnetometers: Addressing Dead Time and Bandwidth for the Unshielded Magnetorelaxometry of Magnetic Nanoparticles

**DOI:** 10.3390/s21041212

**Published:** 2021-02-09

**Authors:** Aaron Jaufenthaler, Thomas Kornack, Victor Lebedev, Mark E. Limes, Rainer Körber, Maik Liebl, Daniel Baumgarten

**Affiliations:** 1 Institute of Electrical and Biomedical Engineering, UMIT—Private University for Health Sciences, Medical Informatics and Technology, 6060 Hall in Tirol, Austria; daniel.baumgarten@umit-tirol.at; 2 Twinleaf LLC, Plainsboro Township, NJ 08536, USA; kornack@twinleaf.com (T.K.); mlimes@twinleaf.com (M.E.L.); 3 Department Biosignals, Physikalisch-Technische Bundesanstalt (PTB), 10587 Berlin, Germany; victor.lebedev@ptb.de (V.L.); rainer.koerber@ptb.de (R.K.); maik.liebl@ptb.de (M.L.); 4 Institute of Biomedical Engineering and Informatics, Technische Universität Ilmenau, 98693 Ilmenau, Germany

**Keywords:** optically pumped magnetometer, free-precession decay, pulsed magnetometer, magnetic nanoparticles, magnetorelaxometry, unshielded, low dead time, high bandwidth, portable setup

## Abstract

Magnetic nanoparticles (MNP) offer a large variety of promising applications in medicine thanks to their exciting physical properties, e.g., magnetic hyperthermia and magnetic drug targeting. For these applications, it is crucial to quantify the amount of MNP in their specific binding state. This information can be obtained by means of magnetorelaxometry (MRX), where the relaxation of previously aligned magnetic moments of MNP is measured. Current MRX with optically pumped magnetometers (OPM) is limited by OPM recovery time after the shut-off of the external magnetic field for MNP alignment, therewith preventing the detection of fast relaxing MNP. We present a setup for OPM-MRX measurements using a commercially available pulsed free-precession OPM, where the use of a high power pulsed pump laser in the sensor enables a system recovery time in the microsecond range. Besides, magnetometer raw data processing techniques for Larmor frequency analysis are proposed and compared in this paper. Due to the high bandwidth (≥100 kHz) and high dynamic range of our OPM, a software gradiometer in a compact enclosure allows for unshielded MRX measurements in a laboratory environment. When operated in the MRX mode with non-optimal pumping performance, the OPM shows an unshielded gradiometric noise floor of about $600\;fT/cm/\sqrt{\mathrm{Hz}}$ for a 2.3 cm baseline. The noise floor is flat up to 1 kHz and increases then linearly with the frequency. We demonstrate that quantitative unshielded MRX measurements of fast relaxing, water suspended MNP is possible with the novel OPM-MRX concept, confirmed by the accurately derived iron amount ratios of MNP samples. The detection limit of the current setup is about 1.37 μg of iron for a liquid BNF-MNP-sample (Bionized NanoFerrite) with a volume of 100 μL.

## 1. Introduction

Magnetic nanoparticles (MNP), particles with a diameter in the nanometer range, offer a large variety of promising applications in medicine. Due to their magnetic core, the particles can be targeted and heated by applying magnetic fields, which is exploited in magnetic hyperthermia and magnetic drug targeting [1]. For these applications, it is crucial to quantify the (spatial) amount of MNP, which can be obtained by means of magnetorelaxometry (imaging) [2]. Furthermore, the retrieval of MNP characteristics and their binding state is fundamental for most biomedical applications. For example, the heating of MNP in magnetic hyperthermia varies with their dynamic properties [3]. In magnetorelaxometry (MRX), the magnetic moments of the MNP are aligned by an external magnetic field, forming a net magnetic moment. After switching this field off, the relaxation of this net moment is commonly detected by a highly sensitive superconducting quantum interference device (SQUID) [4]. The amplitude and the dynamics of this relaxation curve provide quantitative information about the MNP. MNP quantification and spatial quantitative imaging in MRX can be accomplished by analyzing the relaxation amplitudes. The dynamics of MNP magnetic moments can be described by two processes, which occur in parallel. Whole particle rotation is named Brownian relaxation and the rotation of the MNP’s internal magnetization is called Néel relaxation. By analyzing the temporal properties of the relaxation curve, the MNP’s binding state can be obtained [5]. The zero field Néel relaxation time constant is defined as [6]:

(1) $$\tau_{N}=\tau_{0}\exp\left(\frac{KV_{c}}{k_{B}T}\right),$$

where $\tau_{0}$ is the damping time, *K* is the effective magnetic anisotropy, $V_{c}$ is the particle’s core volume, $k_{B}$ is the Boltzmann constant and *T* is the temperature. The Brownian relaxation time constant $\tau_{B}$ depends on the viscosity η, the particle’s hydrodynamic volume $V_{h}$ and the temperature *T* [7]:

(2) $$\tau_{B}=\frac{3\eta V_{h}}{k_{B}T}.$$

If both processes occur in parallel, the faster process dominates, resulting in an effective relaxation time constant $\tau_{\mathrm{eff}}$ [8]:

(3) $$\tau_{\mathrm{eff}}=\frac{\tau_{N}\tau_{B}}{\tau_{N}+\tau_{B}}.$$

The net magnetic moment of an MNP ensemble can be described by the superposition of the single MNP’s magnetic moments. The relaxation of the magnetic moment of the MNP ensemble gives rise to a time dependent net magnetic flux density *B* at the sensor location, while assuming equal relaxation times for each MNP in the sample [8]:

(4) $$B\left(t\right)=B_{\mathrm{relax}}\exp\left(-\frac{t}{\tau_{\mathrm{eff}}}\right)+O,$$

with the relaxation amplitude $B_{\mathrm{relax}}$ and the offset *O*. Often, the core and hydrodynamic particle size distribution is well described by the logarithmic normal distribution. Additionally, MNP might form clusters due to aggregation. A (cluster) moment superposition model can be used to describe the relaxation process of real MNP systems, however the fit is an ill-conditioned inverse problem [9,10]. As an alternative, experimental data can be described by the phenomenological parameters ΔB and $t_{1/e}$ [10]. The difference between the first and last measured magnetic field values is the relaxation amplitude ΔB. The time span in which the first measured magnetic field value drops by the factor *e* is called $t_{1/e}$.

While SQUID offer very high sensitivities, and MRX system dead times in the range of 200 μs [4], they require cryogenic cooling. Several other magnetic field sensor principles have been deployed for MRX, like fluxgate magnetometers [11] and giant magnetoresistive sensors (GMR) [12,13]. However, the high precision fluxgate magnetometers are limited by their bandwidth in the lower kHz range [14] and current GMR are limited by their comparably high noise level in the $nT/\sqrt{\mathrm{Hz}}$ range [15]. Although exhibiting high noise, Hall-Effect magnetometers are suitable for MRX experiments at small sensor-target-distances, e.g., in lab-on-a-chip experiments, offering high bandwidths and a dead time of less than 1 μs [16]. There are two modalities of nitrogen-vacancy (NV)-center based magnetometers which can be applied to MNP studies: single nanodiamonds, with a single sensing vacancy, and extended diamond crystals, incorporating a large number of vacancies. The former are an indispensable tool in studies of the microscopic properties of individual MNP [17]. Very recently a variant of the latter was developed, demonstrating the detection of the instantaneous magnetic susceptibility of MNP under a continuous sub-millitesla excitation field [18]. Despite the small possible sample-to-sensor distance, their flexibility in miniaturization and their unprecedented bandwidth, the practically achieved sensitivity only reached 33 nT in a 1 s measurement time, which is roughly five orders of magnitudes worse than, e.g., the sensitivity achieved with the OPM presented in this work.

While optically pumped magnetometers (OPM), measuring the Larmor precession of spin-polarized atoms in a magnetic field have been known for several decades [19,20,21], the discovery of a spin-exchange-relaxation-free (SERF) mode [22] and the availability of solid state lasers allows sensitivities comparable to those of SQUID [23,24]. The developments in OPM technology in the recent years facilitates the use of OPM in magnetorelaxometry [25,26] and especially in magnetorelaxometry imaging [27], while offering a reduced sensor-target distance, flexible positioning and the omission of cryogenic cooling.

While commercially available OPM are suited for MRX of slowly relaxing MNP within well shielded environments [28], due to the limited bandwidth and pump power, OPM usually require a recovery time in the range of a few milliseconds after switching off the external field in the mT range which is required for MNP alignment. So far, this effect prevented the detection of fast relaxing MNP with OPM, e.g., MNP in liquid suspensions with sub-millisecond relaxation time constants. While depending on the operation mode of the OPM, the bandwidth and dynamic range are mostly limited by the linewidth of the resonance, but can be increased by artificially broadening the linewidth (at the expense of losing sensitivity), or by incorporating feedback loops [29]. While OPM based on the free-precession principle are well known [29,30,31,32,33,34,35], recently a bandwidth of up to 400 kHz was demonstrated with sensitivity degrading linearly with frequency [36]. However, in this case the preparation of the atoms is implemented using low power synchronous pumping, which would add to the dead time after switching off the excitation field in MRX. Several other techniques can be applied to establish the detectable Larmor precession of the optically pumped atoms: some of those rely on enforcing the optimal magnetic field configuration for optical pumping [34,35], which however would influence the relaxation behavior of the MNP and are therefore not favorable. This can be further refined by short and weak RF pulses to re-orient the polarized spin and ensure maximal spin detection efficiency [37].

As magnetic shieldings are a crucial factor in terms of cost, availability and flexibility, it is essential to minimize the required shielding for a clinical application. Unshielded MRX has been performed before with SQUID [38] and fluxgates [39], while OPM-MRX has been performed in a weakly shielded environment [26]. Recently, several developments of portable OPM for unshielded operation have been published [40,41,42,43]. It should be noted, that although not commercially available, the high bandwidth sensor presented in [40] is also a well suited candidate for unshielded MRX.

In this work, we investigated the potential of novel, commercially available, high power pulsed-pump beam OPM, by polarizing the system with a short time period and allowing recovery within a few microseconds after switching off the MRX pulse, thereby minimizing the MRX system dead time. This OPM technique has a white noise floor in a bandwidth determined by a chosen shot-to-shot repetition rate (here 1 kHz). Here we show we are able to access in-shot high-frequency signals, with an upper limit determined by the sample rate of the detection (here we use 6.25 MHz), though in this high-frequency regime sensitivity degrades linearly with detection frequency. To compensate for environmental magnetic disturbances, a gradiometric setup is beneficial, which was also employed in this work. Due to the high dynamic range and high bandwidth of this sensor principle, two magnetometers were enclosed in a single compact package, which can be used as a software gradiometer. The results of first MRX measurements of fast relaxing (water suspended) MNP using a pulsed total field OPM are presented, demonstrating that these sensors overcome the current limitations. The measurements were performed in a completely unshielded environment using our developed portable tabletop OPM-MRX setup.

## 2. Materials and Methods

### 2.1. Setup Overview

**Figure 1:**
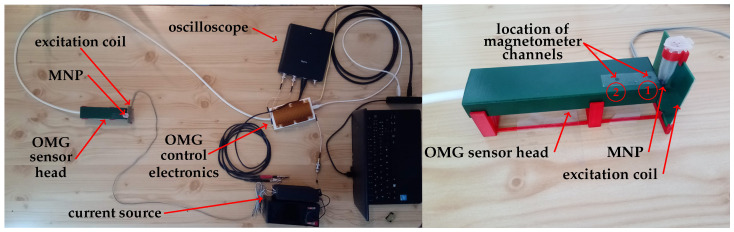
Portable tabletop OPM-MRX setup in unshielded laboratory environment.

Our setup (Figure 1) consists of a single commercially available sensor package containing two separate total field pulsed OPM (OMG—Optical Magnetic Gradiometer from Twinleaf LLC, Plainsboro Township, NJ, USA) and an excitation system for MNP alignment. The unshielded experiments were performed in a laboratory environment, with the Earth’s field as background magnetic field. For the MRX experiments, the MNP samples were placed—one at a time—between the excitation coil and the OMG (Figure 1). The center of the sample is located at a distance of 9.5 mm from the center of the first magnetometer channel.

### 2.2. MNP Excitation Circuit

**Figure 2:**
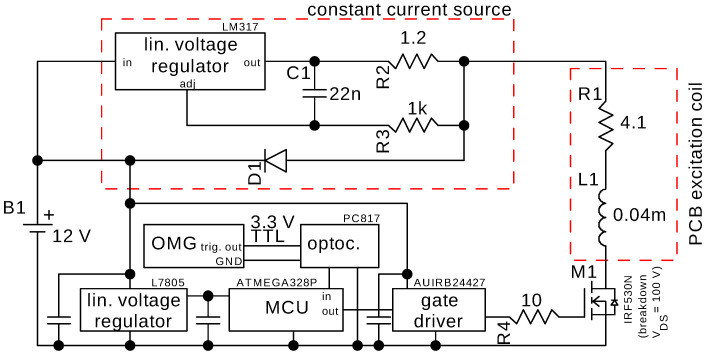
Schematic of a battery-powered coil driver.

A single planar rectangular spiral printed circuit board (PCB) coil (Figure 1) was fabricated and used to align the magnetic moments of the nanoparticles. The schematic of the coil driver is shown in Figure 2. Most critical is the fast current shut-off with minimal ringing. For a fast switch-off, the energy stored in the coil has to be dissipated rapidly. The use of a free wheeling diode, often used to protect electronics from voltage spikes of switched inductive loads, is not recommended for this purpose. The clamping voltage of a standard diode is low, e.g., 0.7 V and so is the dissipated power, leading to a slow shut-off of the magnetic field. An increased clamping voltage can be obtained by the use of a transient voltage suppression (TVS) diode and is limited by the maximum allowed drain-source-voltage of the MOSFET which needs to be protected. Alternatively, an avalanche rated MOSFET can be used [13,44]. Due to the low inductance of the PCB coil of 40 μH and the current of 1.1 A, the stored energy is well within the avalanche limits of the IRF530-MOSFET used here and no additional TVS-diode is required.

While the magnetic field of the PCB coil is not homogeneous, all the samples were placed at the same position. Thus, the inhomogeneity affects all samples in the same manner, allowing the comparison of the measurements. An inhomogeneous excitation field was selected on purpose, as it simplifies the setup and is favorable for MRX imaging [45]. The magnetic flux density at the center of the sample is about 1.4 mT. It should be noted that MNP in a strong magnetic field gradient exhibit directed motion. This is however not the case in this report, since the applied gradients were at least one order of magnitude smaller than needed for a detectable motion.

### 2.3. MNP

For our experiments, dextran based, water suspended magnetic nanoparticles (BNF-Dextran, product code 84-00-801, Micromod Partikeltechnologie GmbH, Rostock, Germany) with a hydrodynamic diameter of 80 nm were used. BNF (Bionized NanoFerrite) particles are widely used in hyperthermia studies for cancer treatment [46,47]. The iron concentration of the factory supplied MNP suspension is specified as 13.7 mg/mL. A dilution series with a sample volume of 100 μL was prepared. The MNP were diluted with distilled water. The dilution factors ranged from 1:1 to 1:1000, resulting in iron amounts of 1.37 mg down to 1.37 μg (Table 1). Additionally, two 100 μL samples filled with Perimag${}^{®}$ (product code 102-00-132, Micromod Partikeltechnologie GmbH, Rostock, Germany) were prepared. The dilution factors were 1:1 and 1:10, resulting in iron amounts of 850 μg and 85 μg. The MNP are multi-core particles, consisting of a cluster of iron oxide crystals with diameters ranging from 3 nm to 8 nm [48]. The mean hydrodynamic diameter is specified as 130 nm. While Brownian relaxation and Néel relaxation occur in parallel, for the MNP used in this work Brownian relaxation is dominant. Our MRX-setup detects the relaxation of the particles with effective time constants in the micro- and millisecond ranges. Due to the exponential decay of the magnetic field over time, the OPM recovery time after the shut-off of the external 1.4 mT magnetic field for MNP alignment is critical.

### 2.4. OPM: Optical Magnetic Gradiometer (OMG)

**Figure 3:**
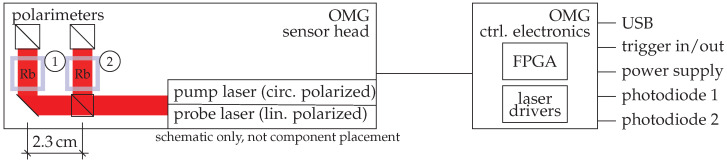
Schematic drawing of the pulsed optically pumped magnetometers (OPM), consisting of two pulsed magnetometers enclosed in a compact sensor head.

The key to a very short OPM dead time is the use of a high intensity, 1 W pump beam laser to rapidly polarize alkali metal atoms in the sensor. The sensor employs two $27\;mm^{3}$ vapor cells with a vapor of rubidium atoms (enriched ^87^Rb). After pumping, the atoms freely precess and their projection is monitored by optical rotation of a linearly polarized probe beam light. The off-resonance 100 μW probe beam light is generated by a single mode, polarization stable vertical-cavity surface-emitting laser (VCSEL). Both, the pump and probe laser are tuned near the 795 nm rubidium resonance manually. The rubidium polarization relaxation rate is dominated by spin-exchange relaxation. With the pump beam shut off for the duration of the measurement a class of systematic errors from pump lightshift to pointing noise are completely eliminated, resulting in a very clean and high precision frequency-based magnetic field measurement. The high power optical pumping substantially resets and erases the time history of the alkali polarization, rendering an independent magnetic field measurement each ms. It should be noted that there is no frequency feedback or resonance tracking as used in other types of self-oscillating magnetometers. This also enhances OPM bandwidth. The different elements of the commercially available sensor are sketched in Figure 3. The sensor is composed of two magnetometers; i.e., it houses two vapor cells. The pump beam and probe beam are split and distributed to the two cells, enabling a future common laser noise reduction as in [49]. The two amplified photodiode signals are available as analog outputs of the OPM control electronics. Additionally, the signals are filtered with a passband between 100 and 500 kHz and are fed into an FPGA inside the OMG control electronics, which measures the frequency and sends the result via USB connection.

### 2.5. Data Acquisition, System Synchronization and Mains Synchronization

The OMG has two analog photodiode outputs, a USB interface and a trigger port. The photodiode signals are digitized by a 16 Bit USB oscilloscope (Handyscope HS6 DIFF from TiePie engineering, The Netherlands) with a sample rate of 6.25 MS/s. The input range was set to ±200 mV for the photodiode channels. The magnetic field measurements of the OMG’s internal FPGA are transferred to a notebook over USB at a sample rate of 1 kHz. The oscilloscope is USB-powered by a battery-powered notebook to prevent injecting 50 Hz noise into the system. The OMG itself is powered from an off-the-shelf 5 V switching-mode power supply.

To synchronize the MRX-coil switch-off with the OMG pump laser, the OMG’s trigger port is configured as output and is fed into the MRX excitation coil electronics. The current monitor of the excitation coil electronics is acquired by the oscilloscope. To take into account the ≈50Hz noise from the Mains power, the MRX sequence length is selected as 30 ms, composed of 10 ms of excitation time and 20 ms of relaxation time. When averaging an even number of MRX sequences, 50 Hz noise will be reduced substantially.

### 2.6. Data Processing: Raw Photodiode Data

**Figure 4:**
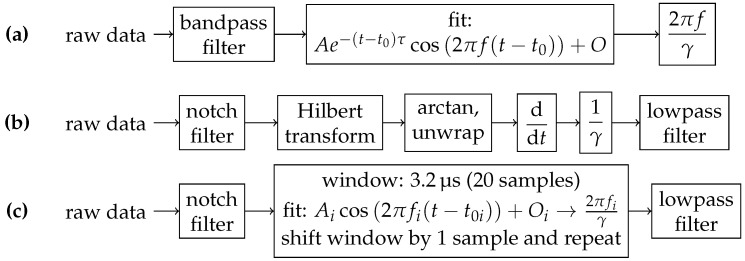
Summary of the raw photodiode processing techniques used: (**a**) free-precession decay fit (1 kHz sample rate); (**b**) Hilbert transform; (**c**) sliding window cosine fit.

As a first step, the raw photodiode data of a single magnetometer channel is split into 1ms chunks, defined by the pump laser pulses. Then the data are processed in three different ways to obtain magnetic field readings. The algorithms used for raw photodiode data processing are summarized in Figure 4.

In the first method, the data are bandpass-filtered to remove electronics noise. The filter used is a 5th order Butterworth with stop frequencies of 290 kHz and 320 kHz, centered at mean Larmor precession frequency of the applied magnetic field (see below). The last 250 μs are discarded, because in this timeslot the electrical heater of the OMG is active. Further discarding of data points due to boundary effects of the filter results in a data snippet of 600 μs length. The data are then fitted to a free-precession decay model using Levenberg-Marquardt algorithm, implemented in SciPy’s *curve_fit*:

(5) $$y\left(t\right)=Ae^{-t/\beta }\cos\left(2\pi f(t-t_{0})\right)+O=Ae^{-t/\beta }\cos\left(\gamma B_{0}\left(t-t_{0}\right)\right)+O,$$

with the amplitude *A*, the decay time constant β, the frequency *f*, phase $t_{0}$ and offset *O*. The obtained frequency *f* is an estimate of the average frequency in the given time period and can be converted to magnetic flux density units by dividing by the gyromagnetic ratio of ^87^Rb (γ/2π=7 Hz/nT). Iterating over each data chunk gives magnetic field readings at a sample rate of 1 kHz.

If a spin ensemble is exposed to a small constant magnetic field $B_{0}$ with a vector component transverse to the pump direction, the precession of the spins occurs at a fixed frequency. Any additional, time varying magnetic field B(t) acts as a frequency modulation of the precession signal:

(6) $$y\left(t\right)=\cos\left(\varphi (t)\right)=\cos\left(\gamma B_{0}t+\gamma \int_{0}^{t}B\left(\tilde{t}\right)d\tilde{t}+\varphi_{0}\right),$$

with the initial phase $\varphi_{0}$. The instantaneous frequency is therefore defined as:

(7) $$f_{\mathrm{inst}}\left(t\right)=\frac{1}{2\pi }\frac{d\varphi (t)}{dt}=\frac{\gamma }{2\pi }\left(B_{0}+B\left(t\right)\right).$$

The phase-estimation is a critical step in the procedure and several algorithms have been proposed [50,51]. While (e.g., after signal normalization) simply applying arccos might be the direct approach, the phase-unwrapping in this case is tricky [52]. Another well known approach is the instantaneous phase estimation via Hilbert transform. The Hilbert transform H can be interpreted as a $90^{\circ }$ phase shifter. The instantaneous phase can therefore be calculated by unwrapping $\arctan\left(\frac{H(y(t))}{y(t)}\right)$. When calculating the Hilbert transform numerically, some limitations have to be known: The modulation frequency must be smaller than the precession frequency at $B_{0}$, otherwise generated frequency sidebands are discarded. However, by separate treatment they still can be demodulated [36]. Typical Hilbert transform implementations are realized as digital filter or incorporate a Fourier transform. Therefore, distortions of the estimated phase near the data-boundaries arise. There are several ways to account for this, e.g., by padding/extrapolating the raw data with, e.g., a mirrored version of the raw signal. However, in this work no padding is used, but the first data points are discarded (as described in the following). This could be addressed in future improvements.

For the high sample rate magnetic field estimation, the photodiode data cannot be filtered with a narrow bandpass, as this would suppress free precession decay sidebands, generated by the frequency modulation. Therefore, magnetic signals with frequencies larger than half the filter width would be discarded. However, the electronics noise has to be filtered prior magnetic field estimation, to not appear in the demodulated signal. A notch filter at 500 kHz was applied. After estimating the instantaneous phase, the derivative and thus the instantaneous frequency is computed. The instantaneous frequency is then lowpass-filtered using a 5th order Besselfilter with a stop frequency of 100 kHz. The first 15 μs are discarded because of boundary effects of the Hilbert transform and the filter.

To ensure the plausibility of the instantaneous frequency method, a sliding window cosine fit with 3.2 μs (20 samples) window width is performed in addition. The fit function is of the form $y\left(t\right)=A\cos\left(2\pi f(t-t_{0})\right)+O$.

This procedure is repeated for the second magnetometer channel. The difference of the obtained magnetometer signals is divided by the baseline (2.3 cm) to obtain the first order gradiometric signal.

The lower boundary of the statistical precision of an unbiased frequency estimator is known as the Cramér-Rao lower bound (CRLB). Assuming a constant magnetic field $B_{0}$ and an additive white Gaussian noise on a free-precession decay signal (Equation (5)), the lower boundary of the frequency estimator’s standard deviation $\sigma_{f}$ can be calculated:

(8) $$\sigma_{f}\geq \frac{\sqrt{12C}}{2\pi \left(A/\rho_{A}\right)T_{s}^{3/2}},C=\frac{N{\left(1-z^{2}\right)}^{3}\left(1-z^{2N}\right)}{12\left(z^{2}{\left(1-z^{2N}\right)}^{2}-N^{2}z^{2N}{\left(1-z^{2}\right)}^{2}\right)},z=e^{-T_{s}/\left(N\beta \right)},$$

with the signal’s spectral noise density at the Larmor frequency $\rho_{A}$, the sampling time $T_{s}$ and the number of samples *N*. The correction factor *C* is applied due to the decaying signal and would be unity for an undamped sine wave. The damping factor β can be estimated from a free-precession decay fit (Equation (5)). The standard deviation $\sigma_{f}$ corresponds to a magnetic noise density of $\rho_{B_{0}}=\sigma_{f}\sqrt{2T_{r}}2\pi /\gamma$, with the repetition time $T_{r}$ [30,53,54]. The achieved sensor performance is compared to this theoretical limit in Section 3.1.

### 2.7. Data Processing: FPGA Data

The 1 kHz data rate measurement stream from the OPM electronics consists of timestamps, absolute field readings from the single magnetometers and the difference of the magnetometers. Besides from dividing the difference-signal by the baseline (2.3 cm) to obtain the first order gradiometric signal, the FPGA data are not filtered or otherwise processed before analyzing the OMG noise floor or the MRX data.

### 2.8. Data Processing: MRX Data

The mean of 100 empty measurements is subtracted from the mean of 100 measured magnetic relaxation signals. As described before, by averaging an even number of sequences with 30 ms length, 50 Hz noise can be reduced substantially. Additionally, the instantaneous magnetic field data obtained via Hilbert transform is lowpass-filtered to trade-off between bandwidth and sensitivity: The first millisecond’s worth of data are 100 kHz lowpass-filtered (see Section 2.6), the second millisecond’s worth of data are 50 kHz lowpass-filtered and the further samples are 30 kHz lowpass-filtered.

Exponential curve fitting to the averaged data is performed using Levenberg-Marquardt algorithm [55] (implemented in SciPy’s *curve_fit*). To account for the MNP diameter distribution or MNP clusters, a phenomenological fit function consisting of the sum of two exponentials is selected:

(9) $$B\left(t\right)=\sum_{i=1}^{2}\left(B_{i}\exp\left(-\frac{t}{\tau_{i}}\right)\right)+O.$$

The relaxation amplitude ΔB and the relaxation time $t_{1/e}$ are computed based on the fit. In MRX experiments, at time 0 s the excitation coil shut-off is initiated. The center of the first precession signal is at 0.466 ms. Therefore, the magnetic field values at timestamps 0.466 ms and 18.466 ms of the fit are used to calculate ΔB. This is done for the FPGA data and for the instantaneous magnetic field data to allow for their comparison.

### 2.9. Proof of Principle Unshielded OPM-MRX with 100 mT Pulsed Fields

**Figure 5:**
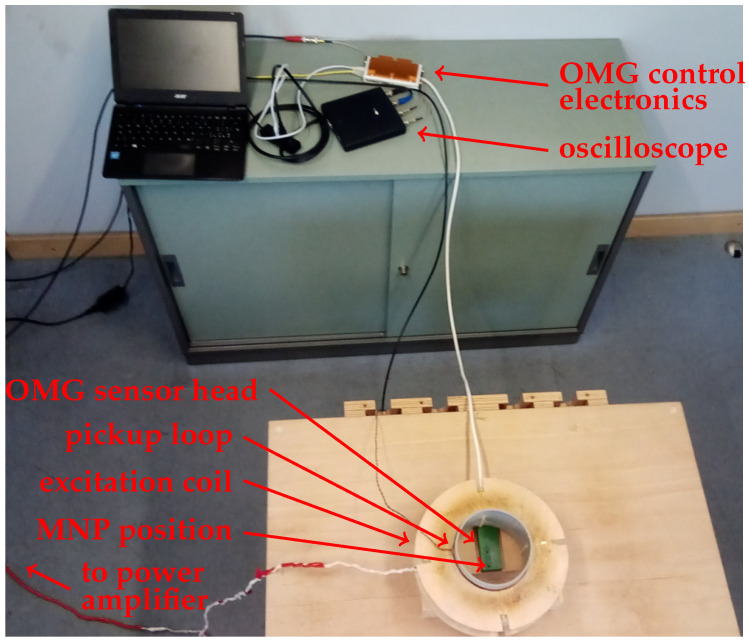
Unshielded OPM-MRX setup with possible MRX excitation fields of up to 100 mT. The battery powered notebook, the USB-oscilloscope and the OMG control electronics are visible on the upper part of the figure. The OMG’s sensor head and the MRX excitation-coil are visible in the lower part of the figure. The position where the MNP sample was later placed is indicated by an arrow. The power amplifier is not visible, as it is located at a distance of about 3 m.

We performed MRX with excitation fields of up to 100 mT to demonstrate that our OPM-MRX setup is not limited to ≈1 mT fields. The excitation coil (Figure 5) was a 304-turn coil with an inner diameter of 125 mm, a resistance of 0.9 Ω and an inductance of 16.2 mH. At its center the coil produces a magnetic flux density of 2.15 mT/A. The coil was powered using a precision power amplifier PA2032A (Rohrer GmbH, Munich, Germany), which is specified to output ±75 V, ±60 A. A fast shut-off of the coil was achieved using solid state switches and a network of TVS-diodes. The switch-off of the excitation coil was monitored by a pickup loop. The OMG was placed underneath the excitation coil and the MNP sample was placed on top of the OMG, at the position of one magnetometer channel (see Figure 5). The MNP sample consisted of a cube of MNP (Berlin Heart GmbH, Berlin, Germany), embedded in gypsum. The gypsum cube with 12 mm edge length was already used in former SQUID-imaging and OPM-MRX-imaging experiments [5,27] and contained a clinically relevant iron concentration of 3.7 mg/cm. The magnetic field values at timestamps 2.44 ms and 120 s of the FPGA data were used to calculate the relaxation amplitude Δ*B*.

## 3. Results and Discussion

### 3.1. OMG Characterization and Performance

**Figure 6:**
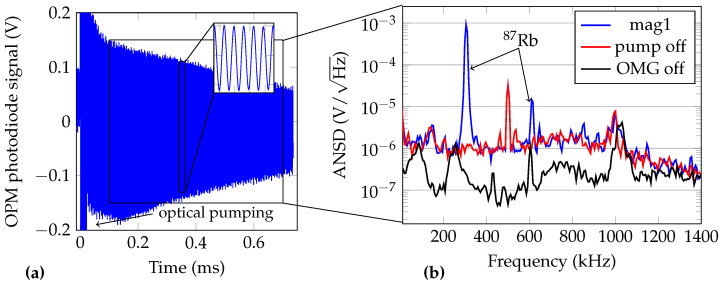
(**a**) Unshielded raw OMG photodiode signal as output by the OPM electronics and recorded using an oscilloscope. (**b**) Amplitude noise spectral densities (ANSD) of the raw OMG analog output: blue—normal operation; red—pump laser always off; black—OMG unpowered. The data used for the calculation of the ANSD were recorded 100 μs to 700 μs after the pump pulse.

One of the OMG’s analog outputs (the amplified photodiode signal), captured by an oscilloscope, is depicted in Figure 6a. The OMG was exposed to environmental noise only. In particular, no MNP were placed in the sample holder and the excitation coil was unpowered.

The signal corresponds to the free spin precession of the alkali atoms. At timestamp 0 s, the pump laser is activated, which saturates the photodiode amplifier. The amplitude spectral density of the precession signal is shown in Figure 6b. Three spectral densities are shown: OMG in normal operation mode; with deactivated pump laser and therefore with no visible polarization rotation; with the OMG completely unpowered. The noise spike at 500 kHz arises from the OPM electronics and not from the switched mode power supply. This was verified using a linearly regulated power supply. The 305 kHz signal component is the free-precession signal of ^87^Rb and corresponds to the Earth’s magnetic field of 43.6 μT. The harmonic at 610 kHz may arise due to orientation-to-alignment conversion due to the linearly polarized probe laser or due to a background magnetic field with a vector component parallel to the pump beam [56,57].

The frequency of the free-precession signal and thus the magnitude of the magnetic field was gathered by multiple methods, as described previously and summarized in Figure 4 (1 kS/s magnetic field data: FPGA detection and free-precession decay fit; 6.25 MS/s magnetic field data: instantaneous frequency extraction using Hilbert transform and sliding window cosine fit).

**Figure 7:**
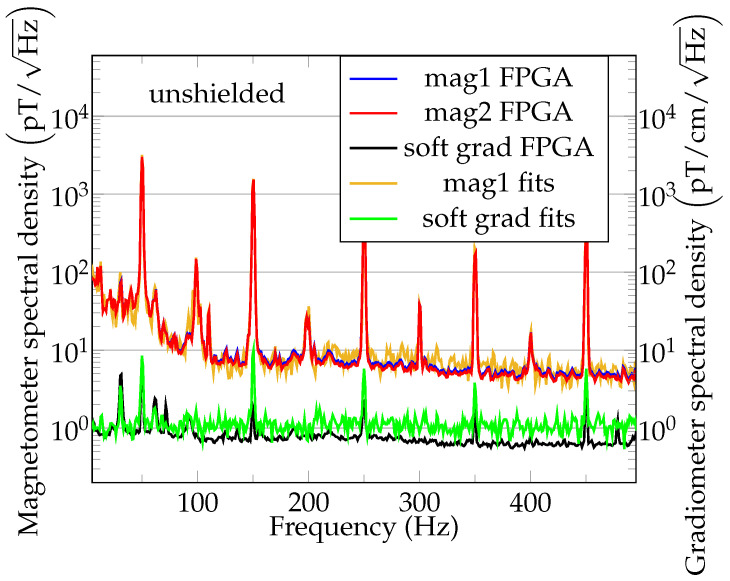
Amplitude noise spectral density (ANSD) of the unshielded OPM in a laboratory environment. The OMG was configured to pump for 22 μs. The software gradiometer baseline is 2.3 cm.

In the case of 1 kS/s magnetic field readings, the data in Figure 6a are converted to one single value of the magnetic field time series. The exemplary unshielded sensor performance with reduced pumping time for MRX is shown in the amplitude noise spectral density (ANSD) plot; see Figure 7. In addition to the FPGA-data noise, the noise floor of the first magnetometer channel in the software gradiometer as estimated from sine-fits (method (a) in Figure 4) is shown in brown/green. The noise level of the FPGA data and fit data is about $5\;pT/\sqrt{\mathrm{Hz}}$ at 500 Hz for the magnetometers. The software gradiometer noise floor of the FPGA data is about $600\;fT/cm/\sqrt{\mathrm{Hz}}$, and it is $1.3\;pT/cm/\sqrt{\mathrm{Hz}}$ for the fit data. The noise floor of the fits is now compared to the CRLB. β=0.80 ms results from a fit of the filtered free precession decay signal to the theoretical model (Equation (5)). Together with the sampling time $T_{s}=600\;\mu s$, this results in C≈2.15. The amplitude A=0.16 V, the spectral noise density $\rho_{A}=0.85\times 10^{-6}\;V/\sqrt{\mathrm{Hz}}$ and the repetition time $T_{r}=1\;ms$ result in a theoretical lower bound of the magnetic noise density of $\rho_{B_{0}}=1.9\;pT/\sqrt{\mathrm{Hz}}$ for the fit data. The theoretical gradiometric noise floor for the fit data scales with $\sqrt{2}$, which gives $1.1\;pT/cm/\sqrt{\mathrm{Hz}}$. As it can be observed in Figure 7, the gradiometric noise floor obtained by the FPGA is lower than the one obtained by curve fitting. The reason is that the curve fitting results are limited by quantization noise of the oscilloscope, while the FPGA readings are of higher precision. An analysis of the FPGA performance can be found in the appendix of [43], while attention has to be paid to the larger baseline and the lower sampling rate used in [43].

**Figure 8:**
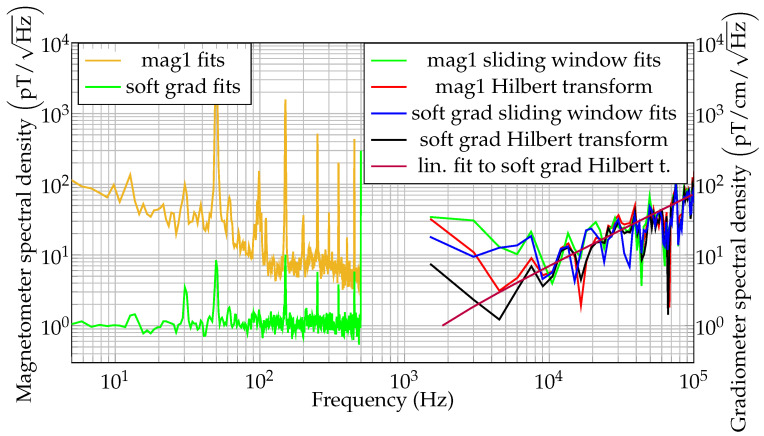
Unshielded magnetometer noise spectral densities using free-precession decay fits (brown), sliding window fits (green) and Hilbert transform (red). Unshielded gradiometer noise spectral densities using free-precession decay fits (green), sliding window fits (blue), Hilbert transform (black) and linear fit to the Hilbert transform (purple).

After notch-filtering the photodiode signal, the instantaneous frequency (IF) at a sample rate of 6.25 MHz is computed using the Hilbert transform. The IF is converted to magnetic field units; thus, the instantaneous magnetic field is obtained. The ANSD of the first magnetometer channel is shown in red in Figure 8, together with the ANSD estimated from sliding window fit magnetic field readings. It should be noted that the low frequency information of the magnetometer spectral density for the high sample rate magnetic field estimations is limited to the lower kHz range. This is due to the repetition rate of the pulsed magnetometer; i.e., in a single cycle only ≈600 μs of data are available, limiting the content of low frequency information. It can be observed that the Hilbert transform and sliding window noise floors match. Additionally, in Figure 8 the gradiometer noise spectral density as calculated by various methods is shown. Please note that the theoretical noise increases linearly with the frequency above 1 kHz. A linear fit to the gradiometric noise spectral density as obtained from the Hilbert transform data is shown in purple in Figure 8. This linear noise increase is typical for free precession decay magnetometers [36], but can also be found in Mx-magnetometers [58]. This can be explained considering Equation (6). The varying magnetic field, e.g., $B\left(t\right)=sin\left(2\pi f_{1}t\right)$, contributes with its integral to the phase φ(t). Therefore, the amplitude of the oscillating part of the phase decreases with $f_{1}$. This decrease is reverted when calculating the phase derivative for obtaining the instantaneous frequency. However, the general system phase noise is white, which is converted to a linearly increasing magnetic field noise floor due to the derivative [36,59]. Note that the magnetometer noise approaches the gradiometer noise for high frequency data, in part due to the decreasing ambient magnetic noise for high frequencies. The observed magnetometer noise floor for unshielded measurements at $B_{0}=43.6\;\mu T$ with a magnetic field sample rate of 100 kHz was about $80\;pT/\sqrt{\mathrm{Hz}}$, while $\approx 80\;pT/cm/\sqrt{\mathrm{Hz}}$ was obtained for the gradiometer. The observed suppression ratio of the environmental 50 Hz noise by the software gradiometer was about 176, as can be observed in Figure 7. Further investigations are needed to assess the noise contributions and suppression performance at higher frequencies (Figure 8).

**Figure 9:**
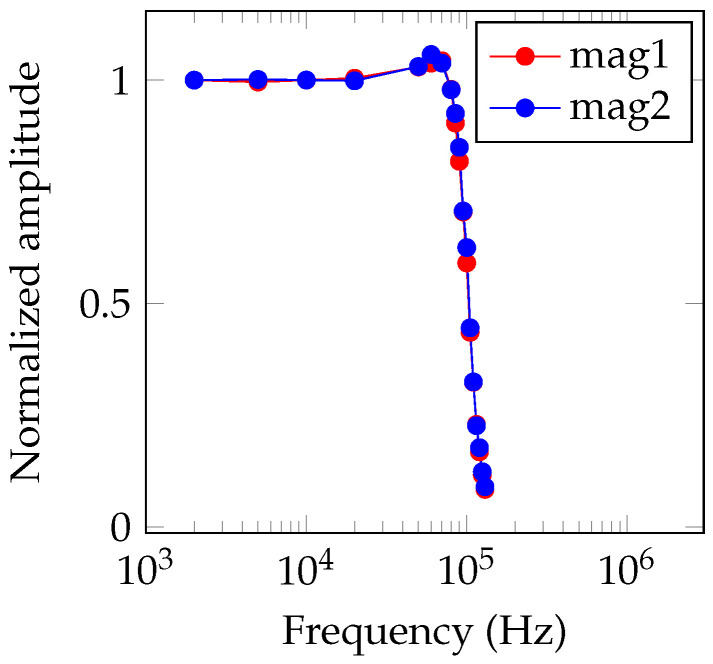
Magnetometer amplitude response over OMG bandwidth for both magnetometer channels using Hilbert transform.

Next, the bandwidth of the OMG in an unshielded environment was estimated by applying a sinusoidally modulated magnetic field with an amplitude 300 nT in the same direction as the background magnetic field. From the bandpass-filtered photodiode signal, the instantaneous magnetic field is calculated using Hilbert transform and the result is 100 kHz lowpass-filtered. Then, a sine wave with fixed frequency (but unknown amplitude and phase) is fitted. The estimated amplitude response is shown in Figure 9 and the estimated bandwidth is 100 kHz, limited by the applied lowpass-filter. The filter’s stop frequency is selected arbitrary and can be increased or decreased, depending on the application (compare also [36]). However, like elaborated before, the increase of the noise floor proportional to the frequency needs to be considered in practical applications.

**Figure 10:**
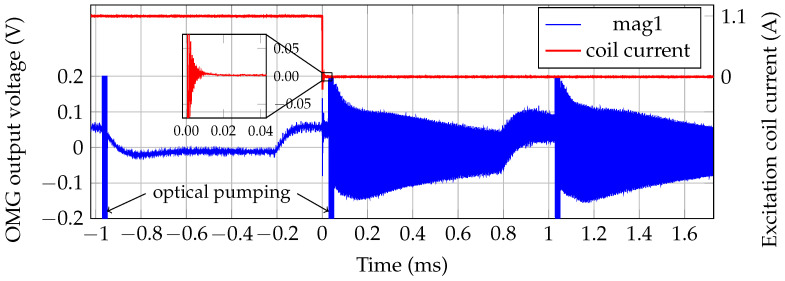
Coil current and OPM photodiode output. The optical pumping started 28 μs after initiating the coil shut-off. The signal distortion prior the pump pulses (i.e., periods −0.2 ms to 0 ms and 0.8 ms to 1 ms) is caused by the electrical heater of the OMG.

In MRX, the sensor dead time after switching off of the excitation coil is a critical parameter. Figure 10 shows the coil current and the photodiode signal of the magnetometer channel close to the excitation coil. It takes approximately 50 μs from the start of the coil switch-off to the end of the OMG’s pumping period. Together with the 15 μs of data discarded during data analysis this results in a system dead time of 65 μs. Therefore, the limits are both, the switch-off and ringing-time of the excitation coil, and the data analysis.

### 3.2. Unshielded MRX with OMG

**Figure 11:**
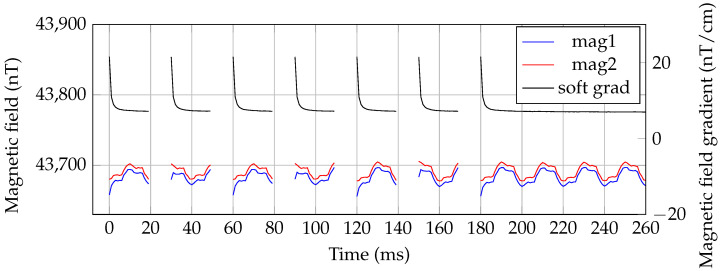
Unshielded OPM-MRX measurements of BNF-MNP (sample with dilution factor 1:20). The excitation coil is on when no FPGA data are available, e.g., in the time span from 20 ms to 30 ms. The FPGA data were not averaged.

In Figure 11, the raw FPGA data of an unshielded MRX-measurement of BNF-MNP (sample with dilution factor 1:20) is shown. The 50 Hz and harmonics disturbations are clearly visible on the single magnetometer channels and are well suppressed with the gradiometric arrangement. A further suppression is reached by averaging.

**Figure 12:**
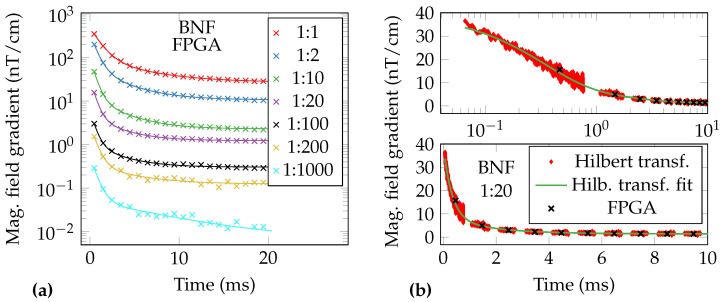
Unshielded OPM-MRX measurements of a BNF-MNP dilution series. The excitation coil was switched off a few μs before the timestamp 0 s. The gradiometric data were averaged 100-times and an averaged empty measurement was subtracted. Individual FPGA-data-points are indicated by crosses, solid lines are the corresponding exponential fits (compare Table 1). (**a**) FPGA data. (**b**) FPGA data and instantaneous magnetic field obtained via Hilbert transform of 1:20 BNF sample; top: logarithmical time axis, bottom: linear time-axis.

After 100-times averaging and subtracting the mean of 100 empty measurements, the resulting MNP relaxation signals are fitted to the double-exponential model (Equation (9)). The FPGA data and the corresponding fits of the dilution series are depicted in Figure 12a. The first FPGA data point is at 0.466 ms after the switch-off of the excitation coil, which is the center of the usable free-precession decay signal. The obtained fit parameters are summarized in Table 1. It should be noted that the static gradient *O* corresponds to the remanence of the MNP [60]. The relation between iron concentration and ΔB of the BNF particles can be described by a linear function with R${}_{\mathrm{adj}}^{2}=0.99$. The increase of the relaxation time $t_{1/e}$ for higher iron concentrations might be due to the resulting increase in viscosity in the samples and interparticle effects [10]. The increase of $t_{1/e}$ for the highly diluted samples might be due to a higher concentration of partially diluted dextran in the samples and therefore the formation of aggregates [10]. The presence of aggregates is supported by the amplitudes and time constants obtained by the double exponential fits (Table 1), where a considerably high second fraction of slower signal contributions is found.

The estimated relaxation time $t_{1/e}$ can be used to calculate a theoretical monodisperse diameter of the MNP (Equation (2)). We assume a sample viscosity equal to that of water. For the 1:20 diluted BNF particles, the relaxation time of 0.62 ms corresponds to a monodisperse diameter of 117 nm, which is in good agreement with the literature [61]. The fit values of the Perimag${}^{®}$ MNP (Table 1) are left as reference. The 1:10 diluted Perimag${}^{®}$ sample’s relaxation time of 1.08 ms corresponds to a monodisperse diameter of 140 nm, which is close to the nominal value from the datasheet 130 nm.

In Figure 12b the averaged FPGA-data of the 1:20 diluted BNF-sample is plotted together with the instantaneous magnetic field as estimated via Hilbert transform and a fit to the double exponential relaxation model (Equation (9)). The estimated fit parameters are shown in Table 1. The Hilbert transform data shown was 100-times averaged and a mean of 100 empty measurements was subtracted. It can be observed, that the high sample rate estimation of the magnetic field obviously suffers from higher noise than the FPGA data, but offers the possibility of examinating the MNP’s relaxation with high time resolution. It is noticeable that the time series data of FPGA and Hilbert transform match very well, except for the first FPGA sample. This is explained by the fact that the FPGA gives a single measurement of the magnetic field over an entire shot, while the magnetic field changes nonlinearly within the first millisecond. The relaxation amplitudes ΔB obtained by the FPGA and the Hilbert transform match. It can be observed that generally shorter relaxation times $t_{1/e}$ are obtained from the Hilbert transform data than from FPGA data. For example, the relaxation time of the 1:20 diluted BNF sample is 0.42 ms, as obtained via Hilbert transform. This corresponds to a monodisperse diameter of 103 nm. Like described before, for the FPGA data a monodisperse diameter of 117 nm is obtained. This confirms that also smaller MNP fractions are detected using the Hilbert transform approach. The estimated relaxation parameters for the other samples are summarized in Table 1. While the fits for samples with lower concentration are still good for the FPGA data, the coefficient of determination R${}_{\mathrm{adj}}^{2}$ of the fits to the Hilbert transform data decreases. This is due to the degradation of the signal-to-noise ratio for low concentrations. For this reason, the fit parameters of the 1:1000 sample are not reported.

### 3.3. Proof of Principle Unshielded OPM-MRX with 100 mT Pulsed Fields

**Figure 13:**
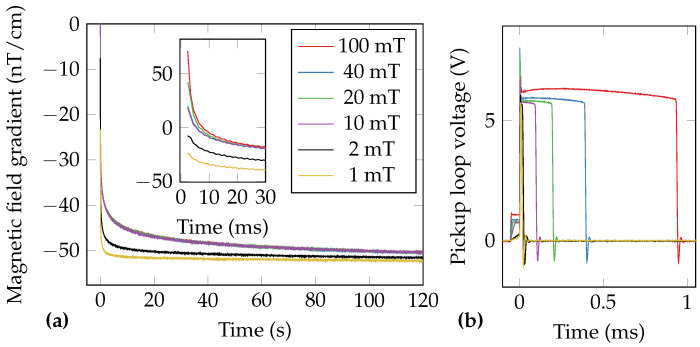
(**a**) FPGA-data of MRX of MNP embedded in gypsum at different excitation fields of up to 100 mT. The inset shows a zoom of the first 30 ms of the relaxations. Please note the different time scale of the figure and inset. The data were not averaged. (**b**) Pickup loop voltage during the shut-off of the different excitation fields.

The reduction in system dead time and the increase of sensor bandwidth leads to the follow-up question regarding the upper limit of the excitation field amplitude. The hand-wound excitation coil (Figure 5) was loaded for 200 ms with currents from 0.5 A to 50 A, resulting in magnetic flux densities at the sample position in the range of 1 mT to 100 mT. It should be noted that the experiment started with the highest excitation field. Special care has to be taken when observing the MRX results, as the coil-shut-off is not immediate and ranges between ≈10 μs for the 1 mT field and ≈1 ms for the 100 mT field (Figure 13b). Therefore, the current decay slope’s effect on the MNP’s relaxation behavior might not be neglectable when analyzing the data on the short time scale (the first few ms). Thus the selection of slowly relaxing MNP for this experiment.

The gradiometric FPGA data of MNP’s embedded in gypsum is shown in Figure 13a. During this experiment, the unshielded gradiometric noise floor was about $1\;pT/cm/\sqrt{\mathrm{Hz}}$. The FPGA data were not averaged or filtered. No empty measurements were subtracted, as the 50Hz gradient is larger than the effects from the excitation coil. Therefore, subtracting non-Mains-synchronized data would result in a slight degradation of the data. The first valid data point is 2.44 ms after initiating the excitation coil switch-off. The excitation coil ringing in the first gradiometer sample is less than 1 nT/cm. The visible static gradient of ≈−50 nT/cm corresponds to the superposition of the MNP sample’s remanence magnetization and the static gradient in the laboratory environment of −11.56 nT/cm. On the large time-scale it can be observed that the MNP are saturated for excitation fields ≥10 mT, which was also observed in SQUID-MRX measurements of the same sample. The estimated relaxation parameters are summarized in Table 2. We would like to mention that a similar non-monotonous effect of the relaxation parameters was observed for Brownian relaxation in [62].

## 4. Conclusions and Outlook

In conclusion, we demonstrated the benefit of pulsed OPM for MRX. Although the principal applicability of OPM for MRX has been shown before [25,26,28], OPM dead time and bandwidth have been prevented from performing MRX on fast relaxing samples, especially with commercially available sensors. To overcome this limit, novel pulsed OPM can be exploited, allowing dead times in the microsecond range and thus the detection of smaller MNP concentrations and the detection of fast relaxing MNP. By exploiting the gradiometric arrangement of our OPM, unshielded MRX in a laboratory environment with a detection limit of 1.37 μg iron with a sample volume of 100 μL was demonstrated. We have shown as a proof of concept that MRX with excitation fields of up to 100 mT is possible with our system.

One important aspect which should be investigated in detail in future work is the channel matching of the magnetometers, which defines the performance of the software gradiometer. Different pump and probe intensities, different temperatures and therefore different buffer gas pressures, different T2 times and photodiode amplifier parameters will directly affect the gradiometer performance and must be studied in future work.

Another point for improvements, which was not focused on in this work, is the data processing of the relaxation curves. To enhance amplitude and time constant parameter estimation, adaptive filtering or resampling [10], or filtering and fitting in the Legendre space [63] should be considered. The latter has the important advantage, that it does not introduce phase shifts like conventional filters.

During the excitation of the MNP most types of SQUID have to be switched to an insensitive mode to avoid saturating the electronics. Therefore, MNP remanence measurements with SQUID is experimentally laborious [60], while fluxgates and total field OPM intrinsically measure the absolute magnetic field. We have shown that the remanence of an MNP sample can be easily obtained with our setup. The remanent magnetization can be exploited in MRX imaging [60]. In this respect it is worth noting that in unshielded experiments, the Earth’s field (or any other bias field) contributes non-neglectably to the remanent magnetization and to the relaxation behavior of the MNP [64,65].

While the OMG’s robustness to high excitation fields might be of interest for studying interparticle effects, this robustness implies the possibility of combining magnetic hyperthermia and MRX in a single setup. Further, imaging of MNP phantoms using MRX with pulsed OPM is envisioned.

## Figures and Tables

**Table 1:** Estimated relaxation amplitudes ΔB, relaxation times $t_{1/e}$ and fit parameters for double exponential fits (Equation (9)) to relaxation curves of liquid BNF MNP and Perimag${}^{®}$ MNP. The values are extracted from FPGA data and from magnetic field readings obtained via Hilbert transform (HT).

Data	MNP	Dilution	Fe	ΔB	$t_{1/e}$	$B_{1}$	$\tau_{1}$	$B_{2}$	$\tau_{2}$	*O*	R${}_{\mathrm{adj}}^{2}$
from	Type	Factor	(μg)	(nT/cm)	(ms)	(nT/cm)	(ms)	(nT/cm)	(ms)	(nT/cm)	
FPGA	BNF	1:1	1370	321.23	1.35	72.57	5.20	389.62	1.10	26.98	1.00
FPGA	BNF	1:2	685	189.21	0.84	45.51	3.51	290.86	0.70	10.71	1.00
FPGA	BNF	1:10	137	44.89	0.64	9.58	3.25	84.19	0.56	2.30	1.00
FPGA	BNF	1:20	68.5	14.44	0.62	3.19	3.05	27.90	0.54	1.23	1.00
FPGA	BNF	1:100	13.7	2.73	0.64	0.74	3.09	4.98	0.54	0.30	1.00
FPGA	BNF	1:200	6.85	1.40	0.72	0.23	4.39	2.48	0.64	0.12	1.00
FPGA	BNF	1:1000	1.37	0.28	0.75	0.05	8.62	0.49	0.65	0.01	1.00
HT	BNF	1:1	1370	265.18	0.18	298.19	1.94	1214.60	0.13	33.42	0.99
HT	BNF	1:2	685	164.52	0.35	120.89	1.86	457.98	0.25	12.00	1.00
HT	BNF	1:10	137	39.68	0.42	21.98	1.81	95.46	0.32	2.64	1.00
HT	BNF	1:20	68.5	12.77	0.42	7.00	1.78	31.17	0.32	1.41	0.99
HT	BNF	1:100	13.7	2.43	0.42	1.52	1.76	5.68	0.31	0.28	0.61
HT	BNF	1:200	6.85	1.25	0.42	0.65	1.92	3.05	0.33	0.10	0.31
FPGA	Perimag${}^{®}$	1:1	850	132.99	1.43	66.40	4.88	128.91	0.81	30.72	1.00
FPGA	Perimag${}^{®}$	1:10	85	16.07	1.08	6.86	4.74	19.18	0.70	3.28	1.00
HT	Perimag${}^{®}$	1:1	850	124.23	0.73	54.94	4.97	191.99	0.49	30.78	1.00
HT	Perimag${}^{®}$	1:10	85	14.96	0.69	9.06	3.56	22.24	0.40	3.44	0.99

**Table 2:** Estimated relaxation parameters ΔB, $t_{1/e}$ and offset *O* of relaxation curves with excitation fields ranging from 1 mT to 100 mT. The sample consists of gypsum-immobilized MNP with a total iron amount of 6.4 mg.

$B_{\mathrm{excitation}}$	ΔB	$t_{1/e}$	*O*
(mT)	(nT/cm)	(ms)	(nT/cm)
100	120.98	6	−50.01
40	69.98	48	−50.09
20	92.34	16	−50.14
10	68.30	58	−50.07
2	43.66	58	−51.24
1	28.65	40	−51.94

## Data Availability

The data presented in this study are available on request from the corresponding author.

## References

[1] Pankhurst Q.A., Connolly J., Jones S.K., Dobson J. (2003). Applications of magnetic nanoparticles in biomedicine. J. Phys. D Appl. Phys..

[2] Richter H., Kettering M., Wiekhorst F., Steinhoff U., Hilger I., Trahms L. (2010). Magnetorelaxometry for localization and quantification of magnetic nanoparticles for thermal ablation studies. Phys. Med. Biol..

[3] Usov N., Liubimov B. (2012). Dynamics of magnetic nanoparticle in a viscous liquid: Application to magnetic nanoparticle hyperthermia. J. Appl. Phys..

[4] Wiekhorst F., Steinhoff U., Eberbeck D., Trahms L. (2011). Magnetorelaxometry assisting biomedical applications of magnetic nanoparticles. Pharm. Res..

[5] Liebl M., Wiekhorst F., Eberbeck D., Radon P., Gutkelch D., Baumgarten D., Steinhoff U., Trahms L. (2015). Magnetorelaxometry procedures for quantitative imaging and characterization of magnetic nanoparticles in biomedical applications. Biomed. Eng. Tech..

[6] Néel L. (1949). Théorie du traînage magnétique des ferromagnétiques en grains fins avec applications aux terres cuites. Ann. Géophys..

[7] Brown W.F. (1963). Thermal fluctuations of a single-domain particle. Phys. Rev..

[8] Raikher Y.L., Shliomis M.I. (1994). The effective field method in the orientational kinetics of magnetic fluids and liquid crystals. Adv. Chem. Phys..

[9] Chantrell R., Hoon S., Tanner B. (1983). Time-dependent magnetization in fine-particle ferromagnetic systems. J. Magn. Magn. Mater..

[10] Eberbeck D., Wiekhorst F., Steinhoff U., Trahms L. (2006). Aggregation behaviour of magnetic nanoparticle suspensions investigated by magnetorelaxometry. J. Phys. Condens. Matter.

[11] Ludwig F., Heim E., Eberbeck D., Schwarz K., Trahms L., Schilling M. (2009). Comparison and Calibration of Fluxgate and SQUID Magnetorelaxometry Techniques for the Characterization of Magnetic Core-Shell Nanoparticles. IEEE Trans. Magn..

[12] Denoual M., Saez S., Kauffman F., Dolabdjian C. (2010). Magnetorelaxometry using Improved Giant MagnetoResistance Magnetometer. Sens. Actuators A Phys..

[13] Zhou X., Huang C.C., Hall D.A. (2017). Giant magnetoresistive biosensor array for detecting magnetorelaxation. IEEE Trans. Biomed. Circuits Syst..

[14] Ludwig F., Heim E., Mäuselein S., Eberbeck D., Schilling M. (2005). Magnetorelaxometry of magnetic nanoparticles with fluxgate magnetometers for the analysis of biological targets. J. Magn. Magn. Mater..

[15] Shen H.M., Hu L., Fu X. (2018). Integrated Giant Magnetoresistance Technology for Approachable Weak Biomagnetic Signal Detections. Sensors.

[16] Liu P., Skucha K., Megens M., Boser B. (2011). A CMOS Hall-effect sensor for the characterization and detection of magnetic nanoparticles for biomedical applications. IEEE Trans. Magn..

[17] Schmid-Lorch D., Häberle T., Reinhard F., Zappe A., Slota M., Bogani L., Finkler A., Wrachtrup J. (2015). Relaxometry and dephasing imaging of superparamagnetic magnetite nanoparticles using a single qubit. Nano Lett..

[18] Kuwahata A., Kitaizumi T., Saichi K., Sato T., Igarashi R., Ohshima T., Masuyama Y., Iwasaki T., Hatano M., Jelezko F. (2020). Magnetometer with nitrogen-vacancy center in a bulk diamond for detecting magnetic nanoparticles in biomedical applications. Sci. Rep..

[19] Dehmelt H. (1957). Modulation of a light beam by precessing absorbing atoms. Phys. Rev..

[20] Bell W.E., Bloom A.L. (1957). Optical detection of magnetic resonance in alkali metal vapor. Phys. Rev..

[21] Dupont-Roc J., Haroche S., Cohen-Tannoudji C. (1969). Detection of very weak magnetic fields (10^−9^gauss) by ^87^Rb zero-field level crossing resonances. Phys. Lett. A.

[22] Happer W., Tang H. (1973). Spin-exchange shift and narrowing of magnetic resonance lines in optically pumped alkali vapors. Phys. Rev. Lett..

[23] Kominis I., Kornack T., Allred J., Romalis M. (2003). A subfemtotesla multichannel atomic magnetometer. Nature.

[24] Allred J.C., Lyman R.N., Kornack T.W., Romalis M.V. (2002). High-Sensitivity Atomic Magnetometer Unaffected by Spin-Exchange Relaxation. Phys. Rev. Lett..

[25] Johnson C., Adolphi N.L., Butler K.L., Lovato D.M., Larson R., Schwindt P.D., Flynn E.R. (2012). Magnetic relaxometry with an atomic magnetometer and SQUID sensors on targeted cancer cells. J. Magn. Magn. Mater..

[26] Dolgovskiy V., Lebedev V., Colombo S., Weis A., Michen B., Ackermann-Hirschi L., Petri-Fink A. (2015). A quantitative study of particle size effects in the magnetorelaxometry of magnetic nanoparticles using atomic magnetometry. J. Magn. Magn. Mater..

[27] Jaufenthaler A., Schier P., Middelmann T., Liebl M., Wiekhorst F., Baumgarten D. (2020). Quantitative 2D magnetorelaxometry imaging of magnetic nanoparticles using optically pumped magnetometers. Sensors.

[28] Baffa O., Matsuda R., Arsalani S., Prospero A., Miranda J., Wakai R. (2018). Development of an Optical Pumped Gradiometric System to Detect Magnetic Relaxation of Magnetic Nanoparticles. J. Magn. Magn. Mater..

[29] Belfi J., Bevilacqua G., Biancalana V., Cartaleva S., Dancheva Y., Khanbekyan K., Moi L. (2009). Dual channel self-oscillating optical magnetometer. JOSA B.

[30] Gemmel C., Heil W., Karpuk S., Lenz K., Ludwig C., Sobolev Y., Tullney K., Burghoff M., Kilian W., Knappe-Grüneberg S. (2010). Ultra-sensitive magnetometry based on free precession of nuclear spins. Eur. Phys. J. D.

[31] Lenci L., Barreiro S., Valente P., Failache H., Lezama A. (2012). A magnetometer suitable for measurement of the Earth’s field based on transient atomic response. J. Phys. B At. Mol. Opt. Phys..

[32] Grujić Z.D., Koss P.A., Bison G., Weis A. (2015). A sensitive and accurate atomic magnetometer based on free spin precession. Eur. Phys. J. D.

[33] Hunter D., Piccolomo S., Pritchard J., Brockie N., Dyer T., Riis E. (2018). Free-induction-decay magnetometer based on a microfabricated Cs vapor cell. Phys. Rev. Appl..

[34] Borna A., Carter T.R., DeRego P., James C.D., Schwindt P.D. (2018). Magnetic source imaging using a pulsed optically pumped magnetometer array. IEEE Trans. Instrum. Meas..

[35] Gerginov V., Pomponio M., Knappe S. (2020). Scalar Magnetometry Below 100 fT/Hz 1/2 in a Microfabricated Cell. IEEE Sens. J..

[36] Wilson N., Perrella C., Anderson R., Luiten A., Light P. (2020). Wide-bandwidth atomic magnetometry via instantaneous-phase retrieval. Phys. Rev. Res..

[37] Li S., Vachaspati P., Sheng D., Dural N., Romalis M.V. (2011). Optical rotation in excess of 100 rad generated by Rb vapor in a multipass cell. Phys. Rev. A.

[38] Haller A., Matz H., Hartwig S., Kerberger T., Atzpadin H., Trahms L. (2001). Low Tc SQUID measurement system for magnetic relaxation immunoassays in unshielded environment. IEEE Trans. Appl. Supercond..

[39] Ludwig F., Mäuselein S., Heim E., Schilling M. (2005). Magnetorelaxometry of magnetic nanoparticles in magnetically unshielded environment utilizing a differential fluxgate arrangement. Rev. Sci. Instrum..

[40] Lebedev V., Hartwig S., Middelmann T. (2020). Fast and robust optically pumped cesium magnetometer. Adv. Opt. Technol..

[41] Oelsner G., IJsselsteijn R., Scholtes T., Krüger A., Schultze V., Seyffert G., Werner G., Jäger M., Chwala A., Stolz R. (2020). Integrated optically pumped magnetometer for measurements within Earth’s magnetic field. arXiv.

[42] Zhang R., Mhaskar R., Smith K., Prouty M. (2020). Portable intrinsic gradiometer for ultra-sensitive detection of magnetic gradient in unshielded environment. Appl. Phys. Lett..

[43] Limes M., Foley E., Kornack T., Caliga S., McBride S., Braun A., Lee W., Lucivero V., Romalis M. (2020). Portable magnetometry for detection of biomagnetism in ambient environments. Phys. Rev. Appl..

[44] Dedman C., Baldwin K., Colla M. (2001). Fast switching of magnetic fields in a magneto-optic trap. Rev. Sci. Instrum..

[45] Crevecoeur G., Baumgarten D., Steinhoff U., Haueisen J., Trahms L., Dupré L. (2012). Advancements in magnetic nanoparticle reconstruction using sequential activation of excitation coil arrays using magnetorelaxometry. IEEE Trans. Magn..

[46] Dennis C., Jackson A., Borchers J., Hoopes P., Strawbridge R., Foreman A., Van Lierop J., Grüttner C., Ivkov R. (2009). Nearly complete regression of tumors via collective behavior of magnetic nanoparticles in hyperthermia. Nanotechnology.

[47] Attaluri A., Kandala S.K., Wabler M., Zhou H., Cornejo C., Armour M., Hedayati M., Zhang Y., DeWeese T.L., Herman C. (2015). Magnetic nanoparticle hyperthermia enhances radiation therapy: A study in mouse models of human prostate cancer. Int. J. Hyperth..

[48] Eberbeck D., Dennis C., Huls N., Krycka K., Grüttner C., Westphal F. (2013). Multicore Magnetic Nanoparticles for Magnetic Particle Imaging. Magn. IEEE Trans..

[49] Schultze V., IJsselsteijn R., Meyer H.G. (2010). Noise reduction in optically pumped magnetometer assemblies. Appl. Phys. B.

[50] Borkowski J., Kania D., Mroczka J. (2018). Comparison of sine-wave frequency estimation methods in respect of speed and accuracy for a few observed cycles distorted by noise and harmonics. Metrol. Meas. Syst..

[51] Harcombe D.M., Ruppert M.G., Fleming A.J. (2020). A review of demodulation techniques for multifrequency atomic force microscopy. Beilstein J. Nanotechnol..

[52] Liu J.L., Zheng J.Y., Wei X.J., Liao F.Y., Luo Y.P. (2018). A new instantaneous frequency extraction method for nonstationary response signals in civil engineering structures. J. Low Freq. Noise Vib. Act. Control.

[53] Cramér H. (1946). Mathematical Methods of Statistics.

[54] Rao C.R. (1992). Information and the accuracy attainable in the estimation of statistical parameters. Breakthroughs in Statistics.

[55] Moré J.J. (1978). The Levenberg-Marquardt algorithm: Implementation and theory. Numerical Analysis.

[56] Rochester S., Ledbetter M., Zigdon T., Wilson-Gordon A., Budker D. (2012). Orientation-to-alignment conversion and spin squeezing. Phys. Rev. A.

[57] Lenci L., Auyuanet A., Barreiro S., Valente P., Lezama A., Failache H. (2014). Vectorial atomic magnetometer based on coherent transients of laser absorption in Rb vapor. Phys. Rev. A.

[58] Vershovskii A., Pazgalev A., Petrenko M. (2020). All-Optical Magnetometric Sensor for Magnetoencephalography and Ultralow Field Tomography. Tech. Phys. Lett..

[59] Li R., Baynes F.N., Luiten A.N., Perrella C. (2020). Continuous High-Sensitivity and High-Bandwidth Atomic Magnetometer. Phys. Rev. Appl..

[60] Baumgarten D., Liehr M., Wiekhorst F., Steinhoff U., Münster P., Miethe P., Trahms L., Haueisen J. (2008). Magnetic nanoparticle imaging by means of minimum norm estimates from remanence measurements. Med. Biol. Eng. Comput..

[61] Remmer H., Dieckhoff J., Schilling M., Ludwig F. (2015). Suitability of magnetic single-and multi-core nanoparticles to detect protein binding with dynamic magnetic measurement techniques. J. Magn. Magn. Mater..

[62] Sarangi S., Tan I., Brazdeikis A. (2011). Brownian relaxation of interacting magnetic nanoparticles in a colloid subjected to a pulsatile magnetic field. J. Nanosci. Nanotechnol..

[63] Bao G., Schild D. (2014). Fast and accurate fitting and filtering of noisy exponentials in Legendre space. PLoS ONE.

[64] Rusakov V., Raikher Y. (2018). Magnetorelaxometry in the Presence of a DC Bias Field of Ferromagnetic Nanoparticles Bearing a Viscoelastic Corona. Sensors.

[65] Jaufenthaler A., Schultze V., Scholtes T., Schmidt C.B., Handler M., Stolz R., Baumgarten D. (2020). OPM magnetorelaxometry in the presence of a DC bias field. EPJ Quantum Technol..

